# Supplementary material for: Unique maternal immune and functional microbial profiles during prenatal stress
Source: Sci Rep. 2020 Nov 20;10:20288. doi: 10.1038/s41598-020-77265-x (PMC7679384; doi:10.1038/s41598-020-77265-x)
Supplement: Supplementary file 2 — Supplementary Information 1. [file 41598_2020_77265_MOESM2_ESM.pdf]

Supplementary Information

Manuscript Title: Unique maternal immune and functional microbial profiles during prenatal stress

Authors: Adrienne M. Antonson, Morgan V. Evans, Jeffrey D. Galley, Helen J. Chen, Therese A. Rajasekera, Sydney M. Lammers, Vanessa L. Hale, Michael T. Bailey, Tamar L. Gur

Supplementary File A. Uploaded as Excel spreadsheet. Metagenomic accession numbers and assembly statistics, MetaPhlAn2 taxonomy, KOFAMScan gene counts, Consensus bin genome copies per million reads in each sample, succinate metabolic gene presence/absence in all MAGs, and GTDBTk taxonomy for both full and consensus MAG sets.

Supplementary Figures:

Supplementary Figure S1.

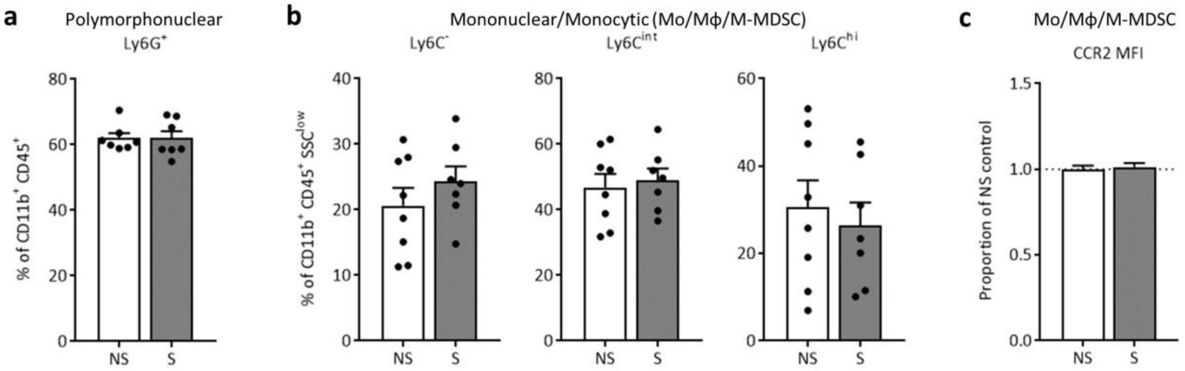

Supplementary Fig. S1. GD11 placental leukocyte populations are protected from prenatal stress. Investigation of GD11 placental tissue indicated that $CD11b^{+}CD45^{+}$ polymorphonuclear (neutrophils or PMN-MDSCs) and SSC^{low} mononuclear (Mo/Mϕ/M-MDSCs) cell populations are highly variable at GD11, and that two days of maternal restraint stress does not shift CCR2 expression in Mo/Mϕ/M-MDSCs. **(a)** $Ly6G^{+}$ neutrophil/PMN-MDSCs, $p = 0.98$. **(b)** Mo/Mϕ/M-MDSCs populations ($Ly6C^{-}$ alternative M2, $p = 0.33$; $Ly6C^{int}$ transitional, $p = 0.70$; and $Ly6C^{hi}$ classical M1, $p = 0.63$). **(c)** CCR2 MFI within Mo/Mϕ/M-MDSCs, $p = 0.65$. Data are mean ± SEM. NS = non-stressed, S = stressed; GD = gestational day; Mo/Mϕ/M-MDSCs = monocytes/macrophages/monocytic myeloid-derived suppressor cells; MFI = median fluorescence intensity.

Supplementary Figure S2.

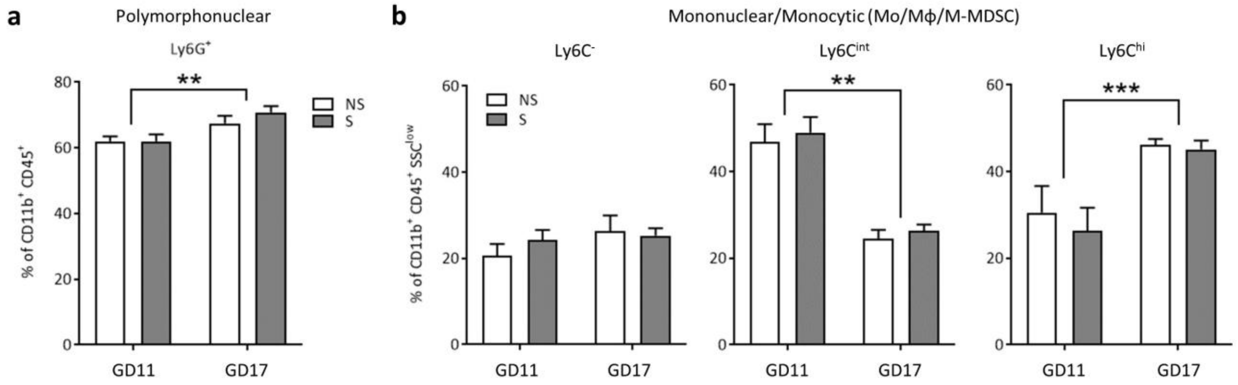

Supplementary Fig. S2. Placental polymorphonuclear and mononuclear populations differed across gestational time points (GD11 vs. GD17). Overall percent of **(a)** placental CD11b^{+}CD45^{+} polymorphonuclear (neutrophils or PMN-MDSCs) cells increased as gestation progressed from GD11 to GD17, regardless of prenatal stress (2-way ANOVA, main effect of gestational day, $p = 0.0015$). Overall percent of **(b)** SSC^{low} mononuclear cells (Mo/Mϕ/M-MDSCs) shifted towards an M1 pro-inflammatory Ly6C^{hi}CCR2^{hi} phenotype (2-way ANOVA, main effect of gestational day, $p = 0.0006$), resulting in a decrease in the Ly6C^{int}CCR2^{int} transitional phenotype (2-way ANOVA, main effect of gestational day, $p < 0.0001$) and no change in alternative M2 Ly6C^{-}CCR2^{-} phenotype (2-way ANOVA, main effect of embryonic day, $p = 0.19$). Data are mean ± SEM. NS = non-stress, S = stress, GD = gestational day, Mo/Mϕ/M-MDSCs = monocytes/macrophages/monocytic myeloid-derived suppressor cells. ** = $p < 0.01$, *** = $p < 0.001$.

Supplementary Figure S3.

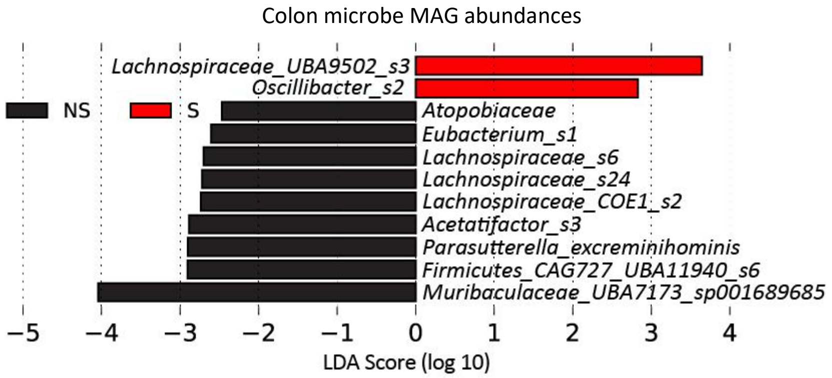

Supplementary Fig. S3. LEfSe plot of differentially abundant metagenome-assembled-genomes (MAGs) in colon content of GD17 dams. LEfSe differential abundance plot, presented as log10 Linear Discriminant Analysis (LDA) effect size score, for MAGs in stressed and non-stressed samples, using genome copies per million reads. Taxonomy is GTDB taxonomy with the lowest possible classification listed. Some MAGs have higher level taxonomy listed for easier identification. GD = gestational day, NS = non-stress (black bars), S = stress (red bars); $p < 0.05$ for all MAGs; $n = 6$/group.

Supplementary Tables:

Supplementary Table S1. Body weight and litter characteristics.

Treatment	Non-stressed Control	N	Stress	N	p-value
Starting weight (g)	21.7 ± 0.27	30	21.8 ± 0.27	34	0.80
GD10 weight (g)	24.9 ± 0.31	30	25.0 ± 0.27	34	0.73
BW gained (g; GD0 to GD10)	3.2 ± 0.17	30	3.2 ± 0.18	34	0.86
Litter size GD11	9.6 ± 0.32	8	9.3 ± 0.29	7	0.45
Fetus resorption #	0.13 ± 0.13	8	0 ± 0	7	0.35
GD16 weight (g)	32.6 ± 0.74	22	31.8 ± 0.40	27	0.36
BW gained (g; GD0 to GD16)	11.1 ± 0.60	22	10.2 ± 0.41	27	0.18
Litter size GD17	7.6 ± 0.46	22	8.1 ± 0.30	27	0.38
Fetus resorption #	0.59 ± 0.17	22	0.52 ± 0.12	27	0.73

Body mass, weight gain, and litter characteristics of non-stressed and stressed gestating dams. Unpaired T tests, using Welch's correction for unequal variances. Data are presented as mean ± SEM.

Supplementary Table S2. Spleen gene expression.

Gene	Non-stressed	Stressed	p-value
IL1B	1.0 ± 0.23	0.95 ± 0.15	0.85
IL6	1.0 ± 0.13	0.85 ± 0.11	0.43
IL10	1.0 ± 0.13	0.93 ± 0.14	0.76
IL23A	1.0 ± 0.20	0.90 ± 0.18	0.74
TNF	1.0 ± 0.14	1.17 ± 0.14	0.45
CCL2	1.0 ± 0.12	0.88 ± 0.04	0.28
TLR2	1.0 ± 0.16	1.32 ± 0.18	0.29
TLR4	1.0 ± 0.14	0.84 ± 0.12	0.43

Relative expression of immune genes in spleens of non-stressed and stressed gestating dams at GD17. Unpaired T tests, using Welch's correction for unequal variances. Data are presented as mean ± SEM; n = 5-10/group.

Supplementary Table S3. Circulating cytokines.

Cytokine (pg/mL)	Non-stressed	Stressed	p-value
IL-1β	2.56 ± 0.56	1.86 ± 0.40	0.32
**IL-5**	**6.69 ± 1.13**	**3.74 ± 0.57**	**0.02 ***
IL-6	13.79 ± 2.10	15.10 ± 2.20	0.68
KC/GRO	98.72 ± 9.46	91.91 ± 3.71	0.51
IL-10	6.93 ± 0.45	6.29 ± 0.56	0.40
TNFα	7.64 ± 0.37	7.70 ± 0.35	0.90

Circulating cytokine levels (pg/mL) in the serum of non-stressed and stressed gestating dams at GD17, measured using MSD® multiplex mouse Proinflammatory Panel 1 assay. Restraint stress decreased circulating levels of Th2/mast cell cytokine IL-5 (an eosinophil and B-cell activator). Unpaired T tests, using Welch's correction for unequal variances. Data are presented as mean ± SEM; n = 12-16/group.

Supplementary Table S4. Uterine gene expression.

Gene	Non-stressed	Stressed	p-value
IL1B	1.0 ± 0.21	0.89 ± 0.14	0.66
IL6	1.0 ± 0.09	1.08 ± 0.12	0.65
IL10	0.81 ± 0.07	0.85 ± 0.08	0.68
IL2	1.0 ± 0.22	0.85 ± 0.21	0.64
CCL2	1.0 ± 0.19	1.02 ± 0.11	0.94
ITGAM	1.0 ± 0.13	0.82 ± 0.11	0.31
TLR4	1.0 ± 0.07	1.02 ± 0.05	0.77
TLR2	1.0 ± 0.08	0.87 ± 0.11	0.52

Relative expression of immune genes in uterine tissue of non-stressed and stressed gestating dams at GD17. Unpaired T tests, using Welch's correction for unequal variances. Data are presented as mean ± SEM; n = 6-10/group.

Supplementary Table S5. Differentially abundant MAGs in colonic contents from GD17 dams.

Metagenome-assembled genome (MAG)	Group	LDA score	p value
d__Bacteria_p__Bacteroidota_c__Bacteroidia_o__Bacteroidales_f__Muribaculaceae_g__UBA7173_s__UBA7173sp001689685	NS	4.041014	0.024975
d__Bacteria_p__Firmicutes_A_c__Clostridia_o__Lachnospirales_f__Lachnospiraceae_g__UBA9502_s__3	S	3.64673	0.037373
d__Bacteria_p__Firmicutes_A_c__Clostridia_o__Oscillospirales_f__Oscillospiraceae_g__Oscillibacter_s__2	S	3.04024	0.037373
d__Bacteria_p__Firmicutes_A_c__Clostridia_o__Lachnospirales_f__Lachnospiraceae_g__Acetatifactor_s__3	NS	2.968175	0.010406
d__Bacteria_p__Proteobacteria_c__Gammaproteobacteria_o__Burkholderiales_f__Burkholderiaceae_g__Parasutterella_s__Parasutterellaexcrementihominis	NS	2.942239	0.006485
d__Bacteria_p__Firmicutes_A_c__Clostridia_o__Lachnospirales_f__Lachnospiraceae_g__Eubacterium_J_s__1	NS	2.94131	0.037373
d__Bacteria_p__Firmicutes_A_c__Clostridia_o__4C28d_15_f__CAG_727_g__UBA11940_s__6	NS	2.916534	0.010406
d__Bacteria_p__Firmicutes_A_c__Clostridia_o__Lachnospirales_f__Lachnospiraceae_g__COE1_s__2	NS	2.820463	0.037373
d__Bacteria_p__Firmicutes_A_c__Clostridia_o__Lachnospirales_f__Lachnospiraceae_g___s__24	NS	2.811841	0.037373
d__Bacteria_p__Firmicutes_A_c__Clostridia_o__Lachnospirales_f__Lachnospiraceae_g___s__6	NS	2.751868	0.006485
d__Bacteria_p__Actinobacteriota_c__Coriobacteriia_o__Coriobacteriales_f__Atopobiaceae_g___s__	NS	2.733864	0.037373

Full taxonomy (using GTDB-TK v.1.0.2) of MAGs identified through LEfSe to be differentially abundant in colonic contents of GD17 dams in non-stress or stress groups, with raw LDA score (effect size) and p-value. GD = gestational day, NS = non-stress, S = stress.

Supplementary Table S6. Intestinal gene expression.

Gene	Tissue	Non-stressed	Stressed	p-value
		**Barrier Integrity**		
CLDN1	Colon	1.0 ± 0.21	1.11 ± 0.23	0.73
	Ileum	1.0 ± 0.27	1.10 ± 0.18	0.78
CLDN2	Colon	1.0 ± 0.07	0.93 ± 0.08	0.54
	Ileum	1.0 ± 0.08	1.19 ± 0.10	0.16
CLDN5	**Colon**	**1.0 ± 0.13**	**0.56 ± 0.07**	**0.04 ***
	Ileum	1.0 ± 0.18	0.65 ± 0.10	0.13
MUC2	Colon	0.83 ± 0.15	1.18 ± 0.33	0.35
	Ileum	1.0 ± 0.21	1.03 ± 0.13	0.92
TFF3	Colon	1.0 ± 0.13	1.04 ± 0.17	0.86
	Ileum	1.0 ± 0.21	1.01 ± 0.13	0.98
DEFA1	**Colon**	**1.0 ± 0.21**	**0.46 ± 0.09**	**0.01 ***
	Ileum	0.63 ± 0.15	0.66 ± 0.11	0.87
		**Immune Response**		
IL6	Colon	1.0 ± 0.16	1.13 ± 0.21	0.65
	Ileum	1.0 ± 0.31	1.13 ± 0.47	0.81
IL1B	Colon	1.0 ± 0.10	0.88 ± 0.17	0.53
	Ileum	1.0 ± 0.19	0.97 ± 0.25	0.93
TNF	Colon	1.0 ± 0.20	0.94 ± 0.23	0.86
	Ileum	1.0 ± 0.24	1.08 ± 0.31	0.84
IL10	Colon	1.0 ± 0.23	1.0 ± 0.24	0.99
	Ileum	1.0 ± 0.31	1.64 ± 0.77	0.45
IL23A	Colon	1.0 ± 0.21	1.06 ± 0.25	0.85
	Ileum	1.0 ± 0.20	0.92 ± 0.13	0.74
CCL2	Colon	1.0 ± 0.19	1.18 ± 0.29	0.62
	Ileum	1.0 ± 0.24	1.47 ± 0.58	0.47
TLR2	Colon	0.8 ± 0.13	1.07 ± 0.20	0.31
	Ileum	1.0 ± 0.27	0.96 ± 0.33	0.93
TLR4	Colon	0.67 ± 0.08	1.29 ± 0.31	0.11
	Ileum	1.0 ± 0.22	0.81 ± 0.18	0.50

Relative expression of epithelial barrier and immune-related genes in intestinal tissue of non-stressed and stressed gestating dams at GD17. Unpaired T tests, using Welch's correction for unequal variances. Data are presented as mean ± SEM; n = 7-8/group; bold font and * = $p < 0.05$.

Supplementary Table S7. Gene Primers.

Gene	Assay ID[a]
RPL19	Mm02601633_g1
CRH	Mm01293920_s1
NR3C1	Mm00433832_m1
IL1B	Mm00434228_m1
IL2	Mm00434256_m1
IL6	Mm00446190_m1
IL10	Mm01288386_m1
IL23A	Mm00518984_m1
ITGAM	Mm00434455_m1
TNF	Mm00443258_m1
CCL2	Mm00441242_m1
TLR2	Mm01213946_g1
TLR4	Mm00445273_m1
CLDN1	Mm00516701_m1
CLDN2	Mm00516703_s1
CLDN5	Mm00727012_s1
MUC2	Mm01276696_m1
TFF3	Mm00495590_m1
DEFA1	Mm02524428_g1

[a] Applied Biosystems TaqMan Gene Expression Assay identification number.
